# Los patrones electroforéticos de proteínas salivales permiten diferenciar los grupos transandino y cisandino de las especies de *Rhodnius* de Colombia

**DOI:** 10.7705/biomedica.4992

**Published:** 2020-06-30

**Authors:** Arlid Meneses, Cristian Camilo Rodríguez, Yazmín Suárez, Julio César Carranza, Gustavo Adolfo Vallejo

**Affiliations:** 1 Laboratorio de Investigaciones en Parasitología Tropical, Universidad del Tolima, Ibagué, Colombia Laboratorio de Investigaciones en Parasitología Tropical Universidad del Tolima Ibagué Colombia

**Keywords:** Rhodnius, proteínas y péptidos salivales, electroforesis en gel de poliacrilamida, Rhodnius, salivary proteins and peptides, electrophoresis, polyacrylamide gel

## Abstract

**Introducción.:**

Las especies *Rhodnius* (Hemiptera: Reduviidae: Triatominae) están conformadas por insectos hematófagos vectores de *Trypanosoma cruzi*, agente etiológico de la enfermedad de Chagas, y *T. rangeli*, parásito infectivo pero no patógeno para el vertebrado. El estudio de la diversidad proteica de la saliva de estos insectos permite la obtención de perfiles electroforéticos unidimensionales característicos de algunas especies de triatominos. Sin embargo, el reporte de los patrones electroforéticos de proteínas salivales de las especies de *Rhodnius* ha sido escaso.

**Objetivo.:**

Hacer un análisis comparativo de los perfiles electroforéticos unidimensionales de las proteínas salivales de *R. colombiensis, R. pallescens, R. pictipes, R. prolixus* y *R. robustus*.

**Materiales y métodos.:**

Se obtuvieron los perfiles electroforéticos de la saliva de las especies en estudio mediante electroforesis en gel de poliacrilamida con dodecilsulfato sódico (*Sodium Dodecyl Sulfate Polyacrylamide Gel Electrophoresis*, SDS-PAGE) y se construyó un fenograma mediante el método UPGMA (*Unweighted Pair Group Method Using Arithmetic Averages*).

**Resultados.:**

Los perfiles electroforéticos de las proteínas solubles de saliva presentaron bandas en un rango de masa aproximado de 15 a 45 kDa, los cuales permitieron diferenciar las cinco especies estudiadas. El fenograma reveló la existencia de dos grupos principales: uno conformado por los grupos cisandinos Pictipes y Prolixus y otro constituido por el grupo transandino Pallescens.

**Conclusiones.:**

Existen diferencias en los perfiles electroforéticos de las proteínas salivales entre *R. colombiensis, R. pallescens, R. pictipes, R. prolixus* y *R. robustus*, cuya variabilidad permitió construir un fenograma congruente con los grupos del género *Rhodnius*.

## Abstract (en)

**Introduction:**

*Rhodnius* (Hemiptera: Reduviidae: Triatominae) species are made up of haematophagous insect vectors for *Trypanosoma cruzi* (Chagas’ disease aetiological agent) and *T. rangeli*, an infective parasite that is not pathogenic for vertebrate hosts. The study of their salivary protein diversity enables the obtention of characteristic one-dimensional electrophoretic profiles of some triatomine species; however, few reports have dealt with *Rhodnius* species salivary proteins electrophoretic patterns.

**Objective:**

To compare *R. colombiensis*, *R. pallescens*, *R. pictipes*, *R. prolixus,* and *R. robustus’* salivary proteins one-dimensional electrophoretic profiles.

**Materials and methods:**

SDS-PAGE was used for obtaining electrophoretic profiles of saliva from the species under study. The unweighted pair group method with arithmetic mean (UPGMA) was used for constructing a phenogram.

**Results:**

Electrophoretic profiles of soluble saliva had protein bands ranging from 15 to 45 kDa, thereby enabling the five species studied to be differentiated. The phenogram revealed two main groups, one formed by the Pictipes and Prolixus cis-Andean groups and another consisting of the Pallescens trans-Andean group.

**Conclusion:**

Differences were revealed regarding *R. colombiensis*, *R. pallescens*, *R. pictipes*, *R. prolixus,* and *R. robustus* electrophoretic profiles of salivary proteins; their variability facilitated constructing a phenogram which was taxonomically congruent with the groups from the genus *Rhodnius.*

El género *Rhodnius* está compuesto por 21 especies de insectos hematófagos, muchas de ellas indistinguibles morfológicamente entre sí ^1^^,^^2^. Todas las especies de este género son vectores de *Trypanosoma cruzi,* agente causal de la enfermedad de Chagas, y de *T. rangeli,* el cual es infectivo, pero no patógeno para los vertebrados ^3^^,^^4^. Aunque *T. rangeli* podría encontrarse en el intestino de cualquier triatomino, solo en las especies de *Rhodnius* logran infectar las glándulas salivales. Se les considera como los vectores biológicos de este parásito y se ha observado que algunos de sus genotipos son patógenos para algunas especies de *Rhodnius*^4^.

Debido a sus características morfológicas, de comportamiento, biogeográficas y genéticas, el género *Rhodnius* parece ser un taxón monofilético compuesto por tres grupos: el grupo Prolixus, integrado por *R. barreti, R. dalessandroi, R. domesticus, R. milesi, R. marabaensis, R. montenegrensis, R. nasutus, R. neglectus, R. prolixus* y *R. taquarusuensis;* el grupo Pictipes, conformado por *R. amazonicus, R. brethesi, R. paraensis, R. piptipes, R. stali* y *R. zeledoni,* y el grupo Pallescens, del cual hacen parte *R. colombiensis, R. ecuadoriensis* y *R. pallescens.* Los grupos Prolixus y Piptipes se distribuyen al oriente de la cordillera de los Andes y se les denomina cisandinos, en tanto que el grupo Pallescens se encuentra al occidente de la cordillera de los Andes y recibe el nombre de transandino ^5^^-^^9^.

En estudios previos se ha reportado que con el perfil electroforético de las hemoproteínas (nitroforinas) se pueden diferenciar las especies fenotípicamente similares ^10^^-^^12^. Asimismo, se ha evidenciado que los patrones electroforéticos de las proteínas salivales solubles bajo condiciones desnaturalizantes (SDS- PAGE) permiten diferenciar entre especies de triatominos ^13^^-^^15^. Sin embargo, la información disponible sobre los perfiles electroforéticos unidimensionales de la saliva de las especies de *Rhodnius* es limitada.

Por esta razón, en el presente estudio se hizo un análisis comparativo de los perfiles electroforéticos bajo condiciones desnaturalizantes de proteínas salivales solubles de *R. colombiensis*, *R. pallescens, R. pictipes, R. prolixus* y *R. robustus.*

## Materiales y métodos

Las glándulas salivales se obtuvieron a partir de la disección de ninfas de quinto estadio (N_5_) de colonias de *R. colombiensis, R. prolixus*, *R. pallescens*, *R. pictipes* y *R. robustus* establecidas en el insectario del Laboratorio de Investigaciones en Parasitología Tropical de la Universidad del Tolima (cuadro 1). Se emplearon como mínimo 10 ejemplares de cada especie en cada réplica biológica y ensayo electroforético.

**Cuadro 1:** Colonias de las especies de *Rhodnius* empleadas según su procedencia

Especie	Procedencia	Ambiente de captura
*R. colombiensis*	Chaparral, Tolima	Silvestre
	Coyaima, Tolima	Silvestre
	Ibagué, Tolima	Silvestre
	Icononzo, Tolima	Silvestre
	Líbano, Tolima	Silvestre
*R. pallescens*	San Sebastián de Buenavista, Magdalena	Silvestre
*R. pictipes*	Amazonas, municipio desconocido	Silvestre
*R. prolixus*	Boyacá, municipio desconocido	Doméstico
	Coyaima, Tolima	Doméstico
	Medina, Cundinamarca	Doméstico
	Santander, municipio desconocido	Doméstico
	Sierra Nevada de Santa Marta, Magdalena	Doméstico
	Villanueva, Casanare	Silvestre
*R. robustus*	Puerto Asís, Putumayo	Silvestre
		

Después de la disección, las glándulas se lavaron tres veces en solución fisiológica fría (NaCl 0,9 %), se recolectaron en un microtubo en cama de hielo y se suspendieron de nuevo en solución fisiológica fría en una proporción de 2 µl por cada par de glándulas. Para permitir la extravasación de la saliva, las glándulas se perforaron con un alfiler entomológico, se centrifugaron a 9.000 r.p.m. durante 5 minutos a 4 °C y el sobrenadante se transfirió a un nuevo microtubo. La concentración de proteínas solubles totales se determinó por espectrofotometría mediante el método de Bradford ^16^.

Se empleó 1 µg de proteína salival soluble para cada electroforesis y estas se corrieron en geles de resolución de poliacrilamida al 12 % acoplados a un gel de 5 % de concentración usando el sistema Mini PROTEAN Tetra Cell^™^ (Bio-Rad). El corrido electroforético se hizo a 90 V empleando tampón de Tris- glicina (Tris, 25 mM, glicina, 192 mM, SDS 0,1 % p/v) durante 2 horas. Los geles se colorearon con nitrato de plata ^17^^,^^18^. Con el objetivo de comprobar la reproducibilidad del polimorfismo observado en la saliva de *R. prolixus*, se hicieron seis réplicas biológicas con sus respectivos geles electroforéticos, en tanto que para las demás especies se hicieron tres.

Los geles de SDS-PAGE se digitalizaron empleando un fotodocumentador Gel Doc XR+System^™^ (Bio-Rad). Los pesos moleculares de las bandas de proteínas visualizadas se calcularon con el programa Image Lab, versión 5.2.1, y una matriz de caracteres y taxones se elaboró con base en la presencia o ausencia de bandas para calcular el coeficiente de similitud de Nei-Li-Dice ^19^ y construir un fenograma empleando el método UPGMA.

## Resultados

**Figura 1:**
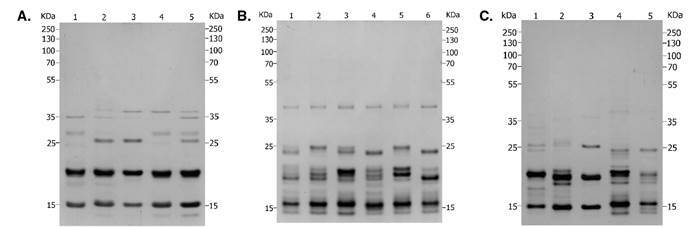
Proteínas salivales en geles de SDS-PAGE al 12 %, coloreados con nitrato de plata. **A)** Especies del género *Rhodnius*: (1) *R. picitipes*, (2) *R. pallescens*, (3) *R. colombiensis* (Coyaima, Tolima), (4) *R. prolixus* (Boyacá), (5) *R. robustus* (Putumayo). **B)** Colonias de *R. prolixus*: (1) Cundinamarca, (2) Tolima, (3) Boyacá, (4) Casanare, (5) Magdalena y (6) Santander. **C)** Colonias de *R. colombiensis*: (1) Ibagué, (2) Icononzo, (3) Coyaima, (4) Chaparral y (5) Líbano

Los perfiles electroforéticos de la saliva de *R. colombiensis*, *R. pallescens*, *R. pictipes*, *R. prolixus* y *R. robustus* mostraron una compleja composición proteica de bajo peso molecular (figura 1), cuyos perfiles fueron similares a los descritos previamente en otras especies de triatominos ^13^^-^^15^, en tanto que las bandas de proteínas tuvieron pesos moleculares menores a 45 KDa, la mayoría de ellas en un rango de 15 a 25 KDa. *Rhodnius pictipes* presentó el mayor número de bandas exclusivas y 22 de ellas eran compartidas, por lo menos, por dos especies. Las poblaciones de *R. prolixus* de Boyacá y Tolima presentaron el mayor número de bandas de proteínas ^14^, y las de *R. colombiensis* de Coyaima y Chaparral presentaron el menor número de bandas (cuadro 2).

**Cuadro 2:** Pesos moleculares en KDa de las bandas de proteínas salivales de cinco especies del género *Rhodnius*

Banda		*R. pictipes*	*R. pallescens*	*R. colombiensis*					R. robustus	*R. prolixus*					
N°	MW			Ibagué	Incononzo	Coyaima	Chaparral	Líbano		Boyacá	Magdalena	Santander	Cundinamarca	Tolima	Casanare
1	42,31 ± 0,37	0	0	0	0	0	0	0	0	1	1	1	1	1	1
2	40,07 ± 0,31	0	0	0	0	0	0	0	1	1	1	1	1	1	1
3	38,03 ± 0,0	1	0	0	0	0	0	0	0	0	0	0	0	0	0
4	37,33 ± 0,38	0	0	0	1	1	1	1	0	0	0	0	0	0	0
5	35,61 ± 0,0	0	1	0	0	0	0	0	0	0	0	0	0	0	0
6	34,49 ± 0,07	0	0	1	1	1	0	1	0	0	0	0	0	0	0
7	32,26 ± 0,0	1	0	0	0	0	0	0	0	0	0	0	0	0	0
8	29,63 ± 0,0	1	0	0	0	0	0	0	0	0	0	0	0	0	0
9	28,19 ± 0,13	0	0	1	1	0	1	1	0	0	0	0	0	0	0
10	27,26 ± 0,0	0	0	0	0	0	0	0	0	0	1	0	0	0	0
11	26,52 ± 0,0	0	1	0	0	0	0	0	0	0	0	0	0	0	0
12	25,89 ± 0,0	1	0	0	0	0	0	0	0	0	0	0	0	0	0
13	25,59 ± 0,01	0	0	0	1	1	0	1	0	0	0	0	0	0	0
14	24,62 ± 0,15	1	0	1	0	0	1	0	1	1	1	0	1	1	0
15	23,8 ± 0,20	0	0	0	0	0	0	0	0	1	1	1	1	1	1
16	23,05 ± 0,18	0	0	0	0	0	0	0	1	1	0	0	0	0	0
17	21,3 ± 0,11	0	0	1	0	0	0	0	0	1	0	0	1	1	1
18	20,35 ± 0,05	0	1	0	0	0	0	0	0	1	1	0	0	0	0
19	19,93 ± 0,13	1	0	0	0	0	0	0	1	1	1	1	1	0	1
20	19,5 ± 0,07	0	1	1	1	1	1	1	0	0	0	0	0	1	1
21	19,1 ± 0,04	0	0	0	0	0	0	0	1	1	0	1	1	1	1
22	18,48 ± 0,08	0	0	0	0	0	0	0	1	1	1	0	0	1	0
23	18,06 ± 0,22	0	1	0	0	0	0	0	0	0	0	1	1	1	1
24	17,68 ± 0,13	1	0	1	1	0	0	0	0	0	0	0	0	0	0
25	17,17 ± 0,0	0	1	0	0	0	0	0	0	0	0	0	0	0	0
26	16,76 ± 0,19	0	0	0	0	0	0	0	1	1	1	0	1	1	1
27	16,23 ± 0,28	1	1	0	0	0	0	0	0	1	1	1	1	1	0
28	15,43 ± 0,06	0	0	0	0	0	0	0	1	1	1	1	1	1	1
29	15,04 ± 0,47	1	1	1	1	1	1	1	1	0	1	1	1	1	1
30	< 15	1	0	0	1	1	1	1	1	1	1	1	1	1	1

MW: *Molecular weight* (peso molecular) en kDa

Significado de los números: 0, banda ausente, 1, banda presente

**Figura 2:**
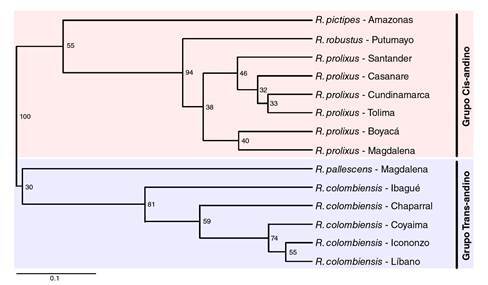
Fenograma construido por el método UPGMA a partir de perfiles electroforéticos de proteínas salivales de cinco especies del género *Rhodnius*. La escala horizontal representa el índice de similitud derivado del índice de Dice.

Con el fenograma construido mediante el método UPGMA se diferenciaron cada una de las especies estudiadas y se evidenció la existencia de dos grupos principales (figura 2): uno conformado por *R. robustus*, *R. prolixus* y *R. pictipes* y, el otro, por *R. pallescens* y *R. colombiensis.*

## Discusión

Los pesos moleculares de las bandas de las proteínas salivales de *R. colombiensis, R. pallescens, R. pictipes, R. prolixus* y *R. robustus* obtenidos mediante SDS-PAGE concordaron con las masas moleculares de las proteínas codificadas por secuencias de ADNc de longitud completa de las glándulas salivales de *R. prolixus*^20^, con algunas proteínas de la familia de las lipocalinas caracterizadas como nitroforinas, con proteínas de unión a amina biogénica y con inhibidores de agregación plaquetaria ^21^^-^^23^, así como con los pesos moleculares de la mayoría de las proteínas identificadas en la saliva de *R. neglectus, R. prolixus*, *R. robustus* y *R. brethesi*^24^^-^^26^. La función principal de estas proteínas es contrarrestar los eventos hemostáticos del huésped, tales como la vasoconstricción, la agregación plaquetaria y la coagulación sanguínea, así como la inflamación y las reacciones del sistema inmunitario del huésped, permitiéndole al insecto un flujo constante de sangre durante su alimentación ^27^^,^^28^.

Estos resultados concuerdan con lo reportado por diferentes autores, quienes mencionan que los perfiles electroforéticos de saliva obtenidos mediante SDS-PAGE de proteínas solubles y electroforesis de hemoproteínas salivales permiten identificar los triatominos hasta el nivel de especie ^10^^-^^12^^,^^14^^,^^15^. La técnica aplicada también puede servir para ayudar a caracterizar las especies del género *Rhodnius* que son morfológicamente similares.

La variabilidad electroforética de las proteínas salivales en la SDS- PAGE en poblaciones de una misma especie, pero proveniente de áreas geográficas diferentes se ha estudiado en *Panstrongylus megistus* de Brasil ^13^ y *Triatoma dimidiata* de Colombia y Guatemala ^14^^,^^15^. Los resultados de estos estudios concuerdan con lo aquí evidenciado en el sentido de que no fue posible establecer una relación entre el polimorfismo observado y el área geográfica o el grado de asociación con las viviendas humanas. No obstante, se han reportado diferencias en los perfiles electroforéticos de hemoproteínas salivales en *R. prolixus* provenientes de Venezuela y la región central de Colombia, lo que evidencia que la variación de la composición proteica puede reflejar distancias geográficas entre las poblaciones de una misma especie ^10^^,^^11^. Es probable que esta relación entre el polimorfismo y la región geográfica pueda observarse al aumentar la escala geográfica y comparar los perfiles electroforéticos de diferentes poblaciones de países de Centroamérica y Suramérica.

Por otra parte, la posición de *R. pictipes* del grupo Pictipes en la misma rama con *R. prolixus* y *R. robustus*, del grupo Prolixus, muestra la cercanía entre los dos grupos; sin embargo, la filogenia de *R. pictipes* y las demás especies del grupo ha sido tema de controversia, ya que debido a su amplia distribución geográfica y a las características ecológicas y las similitudes morfológicas con otros Triatominae y predadores redúvidos no compartidas por la mayoría de las especies de la tribu Rhodniini, *R. pictipes* podría ser la especie más cercana a la forma ancestral del género *Rhodnius*^8^^,^^29^^,^^30^. En estudios de filogenia molecular basados en marcadores nucleares y mitocondriales, los resultados sobre la posición filogenética de *R. pictipes* han diferido. Se ha encontrado que el grupo transandino Pallescens está estrechamente relacionado con el grupo Pictipes ^30^^,^^31^, en tanto que otros autores reportan que *R. pictipes* está más cerca a *R. prolixus* que a las especies del grupo transandino ^1^^,^^32^^,^^33^.

Desde otra perspectiva, en los estudios sobre la interacción de *Rhodnius* y *T. rangeli* se ha postulado una asociación entre los grupos del primero y los genotipos del segundo, observándose que el grupo transandino Pallescens transmite KP1(-) únicamente por inoculación a *T. rangeli,* mientras que el grupo Prolixus solo transmite KP1(+) a *T. rangeli*^4^. Recientemente, se ha reportado que las glándulas salivales de *R. pictipes* pueden ser infectadas por la cepa SC-58 de *T. rangeli* KP1(-). Por lo tanto, *R. pictipes* podría transmitir el genotipo KP1(-) de *T. rangeli*, exhibiendo así características biológicas similares a las evidenciadas en las especies del grupo transandino ^34^.

Puede concluirse que los perfiles electroforéticos de las proteínas salivales permitieron diferenciar entre *R. colombiensis, R. pallescens, R. pictipes, R. prolixus* y *R. robustus,* y que la variabilidad electroforética interespecífica proporcionó datos para la generación de un fenograma congruente con los grupos transandino y cisandino. Asimismo, la información obtenida en este estudio indica que *R. pictipes* puede compartir características fenotípicas con los grupos Pallescens y Prolixus, lo que respalda el estatus de *R. pictipes* como la especie más cercana a la forma ancestral del género *Rhodnius*.
